# Supercapacitor electrode with a homogeneously Co_3_O_4_-coated multiwalled carbon nanotube for a high capacitance

**DOI:** 10.1186/s11671-015-0915-2

**Published:** 2015-05-06

**Authors:** Li Tao, Li Shengjun, Zhang Bowen, Wang Bei, Nie Dayong, Chen Zeng, Yan Ying, Wan Ning, Zhang Weifeng

**Affiliations:** Key Laboratory of Photovoltaic Materials of Henan Province and School of Physics and Electronics, Henan University, Kaifeng, 475001 China; Department of Basic Courses, Yellow River Conservancy Technical Institute, Kaifeng, 475001 China

**Keywords:** Supercapacitor, Cobalt oxide, Multiwalled carbon nanotube, Charge–discharge

## Abstract

Cobalt oxide (Co_3_O_4_) was homogeneously coated on multiwalled carbon nanotube through a simple chemical deposition method and employed in supercapacitor electrodes. SEM image indicated the uniform distribution of Co_3_O_4_ nanoparticles on the surface of the multiwalled carbon nanotube. A maximum specific capacitance of 273 Fg^−1^ was obtained at the charge–discharge current density of 0.5 Ag^−1^. After 500 cycles of continuous charge–discharge process, about 88% of the initial capacity could be retained.

**Electronic supplementary material:**

The online version of this article (doi:10.1186/s11671-015-0915-2) contains supplementary material, which is available to authorized users.

## Background

Electrochemical capacitors (ECs) are causing great concern due to their long cycle life and safety tolerance to high-rate charge and discharge [1]. The electrochemical capacitors have higher power density than secondary batteries and higher energy density than conventional capacitors. With the development of nanoscience and technology, nanoscaled cobalt oxide (Co_3_O_4_) has received great attention for its use in diverse applications such as catalysis, energy storage devices, and electrochemical sensors due to its peculiar properties and controllable morphology compared with the bulk phase [2-4]. In particular, extensive efforts have been devoted to utilize Co_3_O_4_ for supercapacitors because of its high reversibility and theoretical specific capacitance (3560 Fg^−1^) [5,6]. Recently, cobalt oxide has been proven to be a potential alternate to expensive RuO_2_ which is broadly used as the electrochemically active material in electrochemical capacitors [7-12].

It is well known that Co_3_O_4_ is an important p-type semiconductor. Co_3_O_4_ has been used in lithium-ion batteries, heterogeneous catalysis, electrochemical capacitor devices, and other applications. For this purpose, Co_3_O_4_ has been synthesized using a variety of methods such as sol–gel, reflux, microwave, and hydrothermal methods [13-16]. Furthermore, much work has been done on the controlled synthesis of nanostructure Co_3_O_4_ and Co_3_O_4_ cubes, rods, wires, tubes, and sheets [17-21]. Although electrochemical capacitors based on Co_3_O_4_ have shown excellent electrochemical capacity, its practical application in supercapacitors is still limited in part due to its poor electrical conductivity. In order to improve the electrical conductivity, one of the most common ways is to mix the Co_3_O_4_ with conductive additives. Introduction of carbon-based composites may be a promising way to improve the electrical conductivity of Co_3_O_4_. Carbonaceous materials, such as activated carbon, carbon nanotubes (CNTs), and grapheme nanosheets (GNs), can provide matrices for structural stability and fine electron transfer property due to their excellent mechanical flexibility and high electrical conductivity [22-24]. Fu et al. [25] synthesized spherical cobalt oxide nanoparticles along CNTs in supercritical fluid (containing ethanol and CO_2_) and studied their electrical transport properties as a Schottky-junction diode. Huang et al. and Tang et al. obtained hybrid MnO_2_/carbon nanotube through facile redox and hydrothermal methods, respectively, which both showed high-rate capacitility and fine stability [26,27]. Wang synthesized Co_3_O_4_@MWCNT composites through a hydrothermal procedure. This hybrid showed superior electrochemical performance as a cathode material in aqueous supercapacitors, which gave 590 Fg^−1^ at 15 Ag^−1^ in 0.5 M KOH aqueous solution [28]. Su et al. [29] electrodeposited Co_3_O_4_ and NiO on the carbon nanotube and obtained a high capacitance of 52.6 mF cm^−2^.

In this work, we describe a general method to synthesize Co_3_O_4_/MWCNTs through a simple chemical deposition method. Co_3_O_4_ nanoparticles can be evenly and tightly attached on the surface of multiwalled carbon nanotubes (MWCNTs), through a long time of constant temperature heating. The obtained samples showed high specific capacitance (273 Fg^−1^ at a current density of 0.5 Ag^−1^) though just few Co_3_O_4_ was deposited on the MWCNTs. This method could significantly decrease the consumption of rare cobalt element.

## Methods

### Materials preparation

MWCNTs (purity, >95%; diameter, 40 to 60 nm; specific surface area, 200 m^2^g^−1^) were purchased from Chengdu Organic Chemicals Co. Ltd., Chengdu, China. All of the other chemicals were of analytical grade and were used as purchased without further purification. Firstly, MWCNTs were acid-treated with concentrated nitric at 140°C for 10 h. The treated MWCNTs were rinsed with distilled water until the PH was 7 and dried at 60°C for 24 h. It is well known that the surface of MWCNTs possesses a great deal of functional carboxyl groups and becomes negatively charged after functioned with nitric acid [30]. This extraordinary change of the tubular structure for MWCNTs was familiar to be coated with inorganic nanomaterials. Secondly, 80 mg of acid-treated MWCNTs was dispersed into 50 ml ethanol by stirring and ultrasonic treatment, then 2.5 ml of 0.5 M Co(OAC)_2_ aqueous solution was added to the above solution in a state of agitation, followed by the addition of 1 ml of NH_4_OH (30% solution) and 1.4 ml of distilled water an hour later. Thirdly, the reaction was kept at 80°C with stirring for 10 h. After that, the reaction mixture was transferred and sealed in a 100-ml Teflon-lined stainless steel autoclave for a hydrothermal reaction at 150°C for 3 h. After cooling to room temperature, the product was collected by centrifugation and rinsed with deionized water and absolute ethyl alcohol in sequence several times until pH was equal to 7, then dried at 80°C for 12 h. The content of Co_3_O_4_ on the surface of MWCNTs was controlled through the regulation of the Co(OAC)_2_ content. The Co(OAC)_2_ contents were controlled to be 0.125, 0.25, 0.5, and 1 mmol. The prepared samples were denoted as Co_3_O_4_-0.125/MWCNTs, Co_3_O_4_-0.25/MWCNTs, Co_3_O_4_-0.5/MWCNTs, and Co_3_O_4_-1/MWCNTs, accordingly. Pure Co_3_O_4_ sample was also prepared through the same preparation process as the Co_3_O_4_/MWCNTs samples.

### Structural characterization and electrochemical measurements

The morphology and structure of the samples were characterized by JSM-7001 F field emission scanning electron microscope (FESEM) and DX-2700 X-ray diffractometer (XRD) with a monochromatized Cu K irradiation (*k* = 0.154145 nm), respectively. The composition was characterized by the thermogravimetric (TG) analysis method through Netzsch-STA 449C, from 25°C to 900°C at a heating rate of 10°C min^−1^ in air.

The electrochemical measurements were carried out using a three-electrode system with a 6 M KOH electrolyte in which platinum foils and a saturated calomel electrode (SCE) were used as the counter and reference electrodes, respectively. The working electrodes were fabricated by mixing the as-prepared composite, acetylene black, and polytetrafluoroethylene (1% wt.) with a mass ratio of 85:10:5. N-methyl pyrrolidinone (NMP) was also added to form slurry for the spreading on nickel sheets (1.0 cm × 1.0 cm). The working electrodes were pressed at 10 MPa and dried under vacuum at 60°C for 24 h [31]. Cyclic voltammetry (CV) measurement was performed with a CHI660B (Chen Hua Co., Shanghai, China) workstation. The scan rates of CV were in the range from 5 to 100 mVs^−1^. Electrochemical impedance spectroscopy (EIS) measurement was performed with the electrochemistry workstation IM6 (Zahner Co., Kronach, Germany).

## Results and discussion

**Figure 1:**
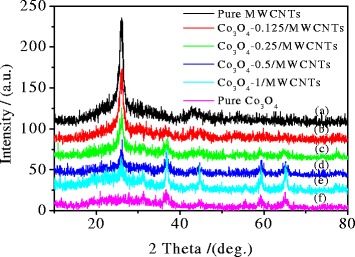
XRD patterns of pure Co_3_O_4_, pure MWCNTs, and Co_3_O_4_/MWCNTs with different Co_3_O_4_ contents. Co_3_O_4_, cobalt oxide; MWCNT, multiwalled carbon nanotube.

XRD patterns of the Co_3_O_4_/MWCNT samples obtained with different cobalt contents are shown in Figure 1. The diffraction peak of the (002) plane for the pure MWCNT sample is sharp. But this peak is gradually weakened with the increasing of Co(OAC)_2_ content, which indicated that the content of Co_3_O_4_ increases accordingly. When the added Co(OAC)_2_ content was 0.25 mmol, new diffraction peaks appeared. These diffraction peaks appeared at 18.9, 31.3, 36.8, 44.9, 59.4, and 65.4°. These peaks belong to the characteristic peaks of spinel Co_3_O_4_, which could be indexed with a JCPDS card (No.43-1003). These diffraction peaks were corresponded to the reflection planes (111), (220), (311), (400), (511), and (440), respectively. These peaks were all in accordance with the pure Co_3_O_4_ nanomaterial, except the peak at 2*θ* = 26°, which is corresponding to the (002) reflection of the MWCNTs. This indicated that the coated Co_3_O_4_ on the surface of MWCNTs has the same crystal phase with the pure Co_3_O_4_ nanoparticles.

**Figure 2:**
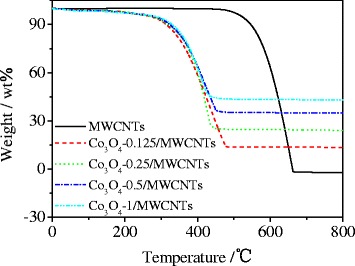
TG curves for the pure MWCNTs and Co_3_O_4_/MWCNT hybrid. MWCNT, multiwalled carbon nanotube.

To quantify the amount of Co_3_O_4_ in the composites, TG analysis was carried out in air. The sample was heated from 25°C to 900°C at a rate of 10°C min^−1^. Figure 2 gives the TG curves for the Co_3_O_4_/MWCNT hybrid and the pure MWCNT powder. It can be seen that the bare MWCNTs are burned off at around 650°C. On the contrary, the Co_3_O_4_-0.5/MWCNT hybrid began to lose the weight significantly at around 400°C due to the catalysis of the Co_3_O_4_ sheath. According to the TG curves, the content of Co_3_O_4_ can be estimated to be about 35% (wt.%) for the Co_3_O_4_-0.5/MWCNTs. The Co_3_O_4_ content of other Co_3_O_4_/MWCNT samples were also decided to be about 13%, 24%, and 43% (wt.%) for the Co_3_O_4_-0.125/MWCNTs, Co_3_O_4_-0.25/MWCNTs, and Co_3_O_4_-1/MWCNTs, respectively. It can be seen that the content of Co_3_O_4_ could be handily controlled through this preparation method.

**Figure 3:**
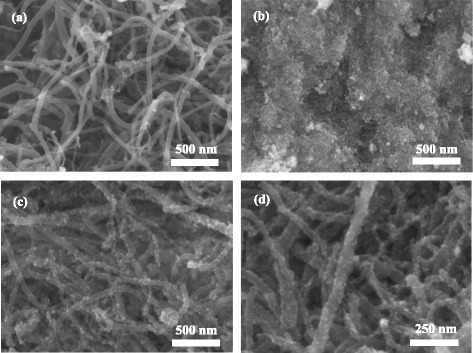
SEM images of **(a)** pure MWCNTs, **(b)** pure Co_3_O_4_, **(c)** Co_3_O_4_-0.5/MWCNTs (low magnification), **(d)** Co_3_O_4_-0.5/MWCNTs (high magnification).

To investigate the surface morphology of Co_3_O_4_/MWCNT composites, SEM measurement was employed. Figure 3 shows the morphologies of the pure MWCNTs (Figure 3a), pure Co_3_O_4_ (Figure 3b), and Co_3_O_4_-0.5/MWCNTs (Figure 3c,d). From Figure 3b, we can see that the as-prepared Co_3_O_4_ particle is small and homogeneous. But the small particles agglomerate with each other to form large powders, which is inconvenient for contact with the electrolyte during the charging and discharging process. It can be seen from Figure 3a that the pure MWCNTs are smooth and flexible, forming strong intertwined entanglements with a three-dimensional (3-D) network structure. Compared with the pure MWCNTs, the surface of Co_3_O_4_-particle-coated MWCNTs (Co_3_O_4_-0.5/MWCNTs) became unsmooth with well-distributed small particles. The particle size is in the range of 5 to 10 nm. The most important thing is that there are no unabsorbed Co_3_O_4_ particles in the samples. It can be seen that the adopted preparation method is a practical way to coat inorganic nanoparticles on MWCNTs. The MWCNTs could provide a conductive frame network for the Co_3_O_4_ electrode for electrochemical capacity. On the other hand, coated Co_3_O_4_ could improve the specific capacity of MWCNT electrode. The SEM spectra of other samples, such as Co_3_O_4_-0.125/MWCNTs, Co_3_O_4_-0.25/MWCNTs, and Co_3_O_4_-1/MWCNTs, are given in the supporting information. It can be seen that the Co_3_O_4_ particles keep increasing with the addition of Co(OAC)_2_ (Additional file 1: Figure S1).

**Figure 4:**
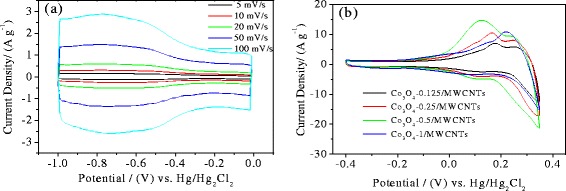
CV curves. CV curves of **(a)** pure MWCNT electrode at different sweep rate, **(b)** Co_3_O_4_/MWCNT electrode with different Co_3_O_4_ contents at a sweep rate of 50 mVs^−1^. Co_3_O_4_, cobalt oxide; MWCNT, multiwalled carbon nanotube.

In order to evaluate the supercapacitor performance of the electrodes, electrochemical studies were performed using cyclic voltammetry in 6 M KOH aqueous electrolyte. Figure 4a illustrates the CV curves of pure MWCNT electrode at different scan rates in the voltage range of −1 to 0 V. The pure MWCNT electrode has deviated from idealized double layer because of the redox reactions of the functional groups on the surface. The paragraph shows high symmetry between the negative curves and the positive ones, so the MWCNT electrode behaves as a pseudocapacitor. With the increase of the sweep rate, the CV curves have no obvious distortion, indicating a highly reversible system.

Figure 4b shows the cyclic voltammograms of Co_3_O_4_/MWCNT composites with different Co_3_O_4_ contents at a scan rate of 50 mV/s between −0.4 and 0.35 V in the 6 M KOH aqueous electrolyte. Compared with the bare MWCNT electrodes, the shapes of the cyclic voltammogram curves in all of the cases are not close to rectangular. The non-rectangular form of the cyclic voltammogram is due to the role of the introduction of Co_3_O_4_ particles which provide higher pseudocapacitive capacity. It is well known that the capacitive behavior of Co_3_O_4_ results from the following redox reactions [32]:

1 $$ \mathrm{C}{\mathrm{o}}_3{\mathrm{O}}_4+\mathrm{O}{\mathrm{H}}^{-}+{\mathrm{H}}_2\mathrm{O}\leftrightarrow 3\mathrm{CoOOH}+{\mathrm{e}}^{-} $$

2 $$ \mathrm{Co}\mathrm{OOH}+\mathrm{O}{\mathrm{H}}^{-}\leftrightarrow \mathrm{C}\mathrm{o}{\mathrm{O}}_2+{\mathrm{H}}_2\mathrm{O}+{\mathrm{e}}^{-} $$

Compared with the pure MWCNT electrode, the Co_3_O_4_-coated MWCNT electrodes show significant higher current density in the CV curves. When the content of added Co(OAC)_2_ was 0.5 M, the obtained samples (Co_3_O_4_-0.5/MWCNTs) give the highest current density which indicated that the Co_3_O_4_-0.5/MWCNT electrode has the highest specific capacitance. When the content of added Co(OAC)_2_ solution was increased to 1 mmol (Co_3_O_4_-1/MWCNTs), the current density decreased abruptly.

**Figure 5:**
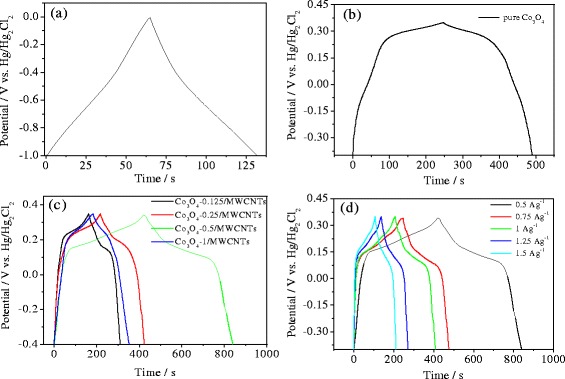
The charge–discharge curves. The charge–discharge curves of the samples **(a)** pure MWCNT electrode at a current of 0.5 Ag^−1^, **(b)** pure Co_3_O_4_ electrode at a current of 0.5 Ag^−1^, **(c)** Co_3_O_4_/MWCNT electrode with different Co contents at a current density of 0.5 Ag^−1^, and **(d)** Co_3_O_4_-0.5/MWCNT electrode at different current density. Co_3_O_4_, cobalt oxide; MWCNT, multiwalled carbon nanotube.

The charge–discharge properties of the electrodes were also tested in 6 M KOH aqueous electrolyte. The charge–discharge curves of pure MWCNT, pure Co_3_O_4_, and Co_3_O_4_/MWCNT composite electrodes are all shown in Figure 5. The charge–discharge current density is 0.5 Ag^−1^. Figure 5a shows the charge–discharge curve of pure MWCNT electrode within a potential window of −1 to 0 V. The shape of the curve is closely linear and shows a typical triangle symmetrical distribution indicating a good double layer capacitive property. Figure 5b shows the charge–discharge curve of the pure Co_3_O_4_ electrode within a potential window of −0.4 to 0.35 V. It can be seen that the curve has significant bend which indicates a pseudocapacitive capacity for the electrodes. Figure 5c shows the charge–discharge of the Co_3_O_4_/MWCNT composites with different cobalt content in the potential range of −0.4 to 0.35 V. The shape of the charge–discharge curves is similar with that of the pure Co_3_O_4_ electrode. The average specific capacitances for the electrodes can be calculated on the basis of Equation 3:

3 $$ C=\left(i \times \Delta t\right)/\left(m\times \Delta V\right) $$

where *C* is the specific capacitance (Fg^−1^), *i* (A) is the discharge current, △*V* (V) is the potential window during the discharge process, △*t* (s) is the discharge time, and *m* (g) is the mass of electroactive material [33]. The average specific capacitances for the pure MWCNT, pure Co_3_O_4_, Co_3_O_4_-0.125/MWCNT, Co_3_O_4_-0.25/MWCNT, Co_3_O_4_-0.5/MWCNT, and Co_3_O_4_-1/MWCNT composite electrodes, which were obtained from charge–discharge curves on the basis of Equation 3, were calculated to be about 33, 150, 103, 137, 273, and 118 Fg^−1^, respectively. The results are identical to that estimated from the cyclic voltammogram curves. This result indicates that it is valuable to combine the high electric conduction of MWCNTs with the large specific capacity of Co_3_O_4_. Meanwhile, the content of the coated Co_3_O_4_ should be appropriate.

Figure 5d displays the charge–discharge curves of the Co_3_O_4_-0.5/MWCNT composite at various current densities in the range of 0.5 to 1.5 Ag^−1^. The specific capacitances were calculated to be about 273, 160, 134, 88, and 69 Fg^−1^, respectively. It can be seen that the specific capacitances gradually decreases with the increasing of the discharging current density. This phenomenon might be due to the diffusion limits of the OH^−^ ion movement.

**Figure 6:**
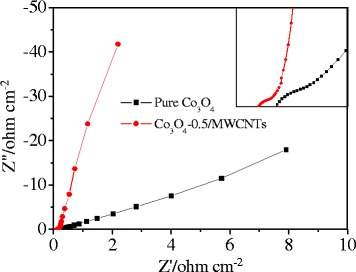
The Nyquist plots of the EIS spectra of Co_3_O_4_-0.5/MWCNTs and pure Co_3_O_4_ electrode. Co_3_O_4_, cobalt oxide; MWCNT, multiwalled carbon nanotube.

The electrochemical performance of the electrode was further investigated by the EIS measurements. Figure 6 shows the Nyquist plots of the EIS spectra of pure Co_3_O_4_ electrode and Co_3_O_4_-0.5/MWCNT electrode, in the frequency range of 1 Hz to 100 KHz. The obtained EIS spectra are composed of a half semicircle at high frequency and a line at low frequency. The small arc observed at the high frequency is related to the process at the electrode material electrolyte interface (*R*_ct_), and the line at the low frequency indicates a capacitive behavior related to the charging mechanism. High-frequency intercepts of the real axis gives the serial resistance (*R*_s_) for the working electrode. It can be seen that the Co_3_O_4_-0.5/MWCNT has smaller *R*_ct_ and *R*_s_, indicating a lower electrochemical reaction resistance and electron transfer resistance. Furthermore, the straight line of the Co_3_O_4_-0.5/MWCNT spectra is more close to 90° compared with the pure Co_3_O_4_ spectra which shows that the Co_3_O_4_-0.5/MWCNTs possess a more ideal capacitive behavior.

**Figure 7:**
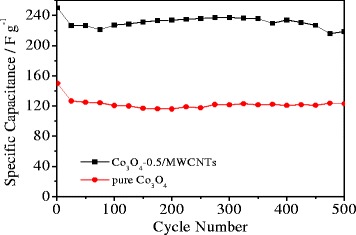
Cycling performances of Co_3_O_4_-0.5/MWCNTs and pure Co_3_O_4_ at current density of 0.5 Ag^−1^. Co_3_O_4_, cobalt oxide; MWCNT, multiwalled carbon nanotube.

Figure 7 shows the cycle life of the pure Co_3_O_4_ and Co_3_O_4_-0.5/MWCNT electrodes at 0.5 Ag^−1^. It is clearly seen that the specific capacitance of the Co_3_O_4_-0.5/MWCNT electrode is much higher than that of pure Co_3_O_4_ under the same current density. The two electrodes show similar charge–discharge performance in the first 500 cycle times. The process of charging and discharging are both relatively stable. After 500 times charge–discharge, the specific capacity of Co_3_O_4_-0.5/MWCNT electrodes remains to be about 219 Fg^−1^ which is about 88% of the first discharge capacity.

## Conclusions

Co_3_O_4_ was homogeneously coated on a multiwalled carbon nanotube through a simple chemical deposition method. The contents of Co_3_O_4_ on the surface of MWCNTs were handily controlled through the regulation of the Co(OAC)_2_ content. Furthermore, the Co_3_O_4_ nanoparticles were homogeneously distributed on the surface of multiwalled carbon nanotubes. The coating of Co_3_O_4_ could significantly increase the specific capacity of MWCNTs. The optimized samples were obtained when the Co(OAC)_2_ content was 0.5 mmol. The maximum specific capacitance of 273 Fg^−1^ was obtained at the charge–discharge current density of 0.5 Ag^−1^. After 500 cycles of charge–discharge process, about 88% of the initial capacity could be retained.

## Additional file

Additional file 1:
**SEM images.** It can be seen that the Co_3_O_4_ particles keep increasing with the addition of Co(OAC)_2_.

## References

[1] Simon P, Gogotsi Y (2008). Materials for electrochemical capacitors. Nature..

[2] Yang G, Gao D, Zhang J, Zhang J, Zhu Z, Xue D (2013). Synthesis and characterization of shape-controlled mesoporous Co_3_O_4_ hierarchical nanostructures. RSC Adv..

[3] Battumur T, Ambade SB, Ambade RB, Pokharel P, Lee DS, Han SH (2013). Addition of multiwalled carbon nanotube and graphene nanosheet in cobalt oxide film for enhancement of capacitance in electrochemical capacitors. Current Appl Phys..

[4] Wang K, Shi ZQ, Wang YY, Ye ZG, Xia HY, Liu GW (2015). Co_3_O_4_ nanowires@MnO_2_ nanolayer or nanoflakes core–shell arrays for high-performance supercapacitors: the influence of morphology on performance. J Alloys Compd..

[5] Yuan C, Yang L, Shen L, Zhang X, Lou XW (2012). Growth of ultrathin mesoporous Co_3_O_4_ nanosheet arrays on Ni foam for high-performance electrochemical capacitors. Energy Environ Sci..

[6] Cheng H, Lu ZG, Deng JQ, Chung CY, Zhang K, Li YY (2010). A facile method to improve the high rate capability of Co_3_O_4_ nanowire array electrodes. Nano Res..

[7] Wei TY, Chen CH, Chang KH, Lu SY, Hu CC (2009). Cobalt oxide aerogels of ideal supercapacitive properties prepared with an epoxide synthetic route. Chem Mater..

[8] Hu CC, Chang KH, Lin MC, Wu YT (2006). Design and Tailoring of the Nanotubular Arrayed Architecture of Hydrous RuO_2_ for Next Generation Supercapacitors. Nano Lett..

[9] Kandalkar SG, Lokhande CD, Mane RS, Han SH (2007). A non-thermal chemical synthesis of hydrophilic and amorphous cobalt oxide films for supercapacitor application. Appl Surf Sci..

[10] Yuan AB, Zhang QL (2006). A novel hybrid manganese dioxide/activated carbon supercapacitor using lithium hydroxide electrolyte. Electrochem Commun..

[11] Pang H, Gao F, Chen Q, Liu RM, Lu QY (2012). Dendrite-like Co_3_O_4_ nanostructure and its applications in sensors, supercapacitors and catalysis. Dalton Trans..

[12] Xiong SL, Yan CZ, Zhang XG, Xi BJ, Qian YY (2009). Controllable synthesis of mesoporous Co_3_O_4_ nanostructures with tunable morphology for application in supercapacitors. Chem Eur J..

[13] Santos GA, Santos CMB, Silva SWD, Urquieta-Gonzalez EA, Sartoratto PPC (2012). Sol–gel synthesis of silica–cobalt composites by employing Co_3_O_4_ colloidal dispersions. Colloids Surf A Physicochem Eng Aspects..

[14] Meher SK, Rao GR (2011). Effect of microwave on the nanowire morphology, optical, magnetic, and pseudocapacitance behavior of Co_3_O_4_. J Phys Chem C..

[15] Bilecka I, Djerdj I, Niederberger M. One-minute synthesis of crystalline binary and ternary metal oxidenanoparticles. Chem Commun. 2008;886. 10.1039/b717334b 18253537

[16] Meher SK, Rao GR (2011). Ultralayered Co_3_O_4_ for high-performance supercapacitor applications. J Phys C..

[17] He T, Chen DR, Jiao XL, Wang YL, Duan YZ (2005). Solubility-controlled synthesis of high-quality Co_3_O_4_ nanocrystals. Chem Mater..

[18] Liu Y, Wang G, Xu C, Wang W. Fabrication of Co_3_O_4_ nanorods by calcination of precursor powders prepared in a novel inverse microemulsion. Chem. Commun. 2002;1486. 10.1039/b202550g 12189856

[19] Li Y, Tan B, Wu Y (2008). Mesoporous Co_3_O_4_ nanowire arrays for lithium ion batteries with high capacity and rate capability. Nano Lett..

[20] Lou XW, Deng D, Lee JY, Feng J, Archer LA (2008). Self-supported formation of needlelike Co_3_O_4_ nanotubes and their application as lithium-ion battery electrodes. Adv Mater..

[21] Cao AM, Hu JS, Liang HP, Song WG, Wan LJ, He XL (2006). Hierarchically structured cobalt oxide (Co3O4): the morphology control and its potential in sensors. J Phys Chem B.

[22] Yang XL, Fan KC, Zhu YH, Shen JH, Jiang X, Zhao P (2012). Tailored graphene-encapsulated mesoporous Co_3_O_4_ composite microspheres for high-performance lithium ion batteries. J Mater Chem..

[23] Lang JW, Yan XB, Xue QJ (2011). Facile preparation and electrochemical characterization of cobalt oxide/multi-walled carbon nanotube composites for supercapacitors. J Power Sourc..

[24] Su L, Jing Y, Zhou Z (2011). Li ion battery materials with core–shell nanostructures. Nanoscale..

[25] Fu L, Liu Z, Han B, Han P, Hu P, Cao L (2005). Beaded cobalt oxide nanoparticles along carbon nanotubes: towards more highly integrated electronic devices. Adv Mater..

[26] Tang W, Hou YY, Wang XJ, Bai Y, Zhu YS, Sun H (2012). Beaded cobalt oxide nanoparticles along carbon nanotubes: towards more highly integrated electronic devices. J Power Sourc..

[27] Huang HJ, Zhang WY, Fu YS (2015). Controlled growth of nanostructured MnO_2_ on carbon nanotubes for high-performance electrochemical capacitors. Electrochim Acta..

[28] Wang XW, Li MX, Chang Z, Yang YQ (2015). Co_3_O_4_@MWCNT nanocable as cathode with superior electrochemical performance for supercapacitors. ACS Appl Interf..

[29] Su F, Lv X, Miao M (2015). High-performance two-ply yarn supercapacitors based on carbon nanotube yarns dotted with Co_3_O_4_ and NiO nanoparticles. Small..

[30] Nethravathi C, Rajamathi M (2008). Chemically modified graphene sheets produced by the solvothermal reduction of colloidal dispersions of graphite oxide. Carbon..

[31] Sahoo S, Karthikeyan G, Nayak GC, Das CK (2011). Electrochemical characterization of in situ polypyrrole coated graphene nanocomposites. Synth Met..

[32] Liao MX, Liu YF, Hu ZH, Yu Q (2013). Novel morphologic Co_3_O_4_ of flower-like hierarchical microspheres as electrode material for electrochemical capacitors. J Alloys Compd..

[33] Devaraj S, Munichandraiah N (2005). High capacitance of electrodeposited MnO_2_ by the effect of a surface-active agent. Electrochem Solid State Lett..

