# Supplementary material for: Supercapacitor electrode with a homogeneously Co3O4-coated multiwalled carbon nanotube for a high capacitance
Source: Nanoscale Res Lett. 2015 May 6;10:208. doi: 10.1186/s11671-015-0915-2 (PMC4437991; doi:10.1186/s11671-015-0915-2)
Supplement: Additional file 1: — SEM images. It can be seen that the Co3O4 particles keep increasing with the addition of Co(OAC)2. [file 11671_2015_915_MOESM1_ESM.doc]

Supercapacitor electrode with a homogeneously Co_3_O_4_ coated multiwalled carbon nanotube for a high capacitance

Li Tao^1^, Li Shengjun^1*^, Zhang Bowen^1^, Wang Bei^1^, Nie Dayong^2^, Chen Zeng^1^, Yan Ying^1^, Wan Ning^1^, Zhang Weifeng^1^

1. Key Laboratory of Photovoltaic Materials of Henan Province and School of Physics and Electronics, Henan University, Kaifeng 475001, China

2. Department of Basic Courses, Yellow River Conservancy Technical Institute, Kaifeng 475001, China

*Corresponding author: Shengjun Li, tel/fax: 86-378-3881602, E- mail: [Lishengjun1011@126.com](mailto:Lishengjun1011@126.com)


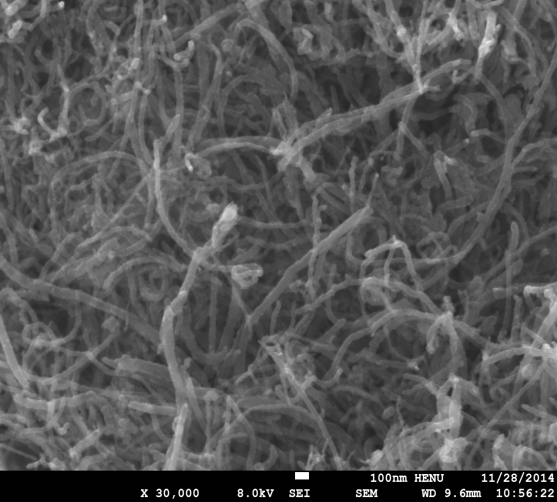


**1 μm**


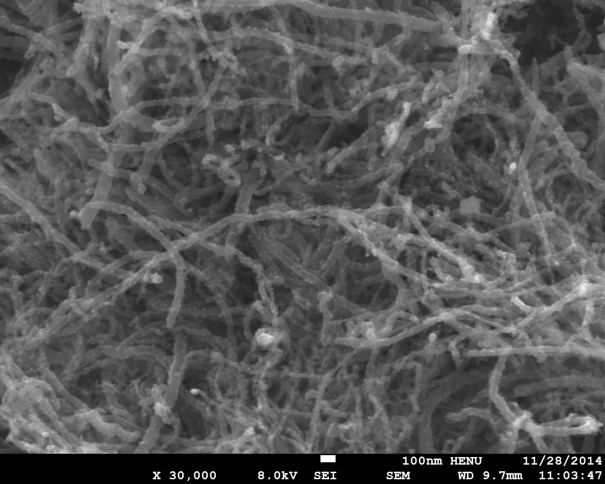


**1 μm**


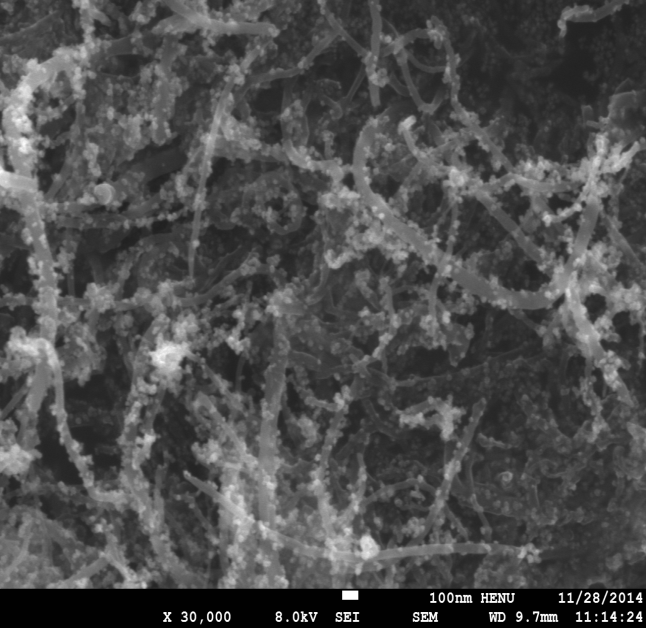


**1 μm**


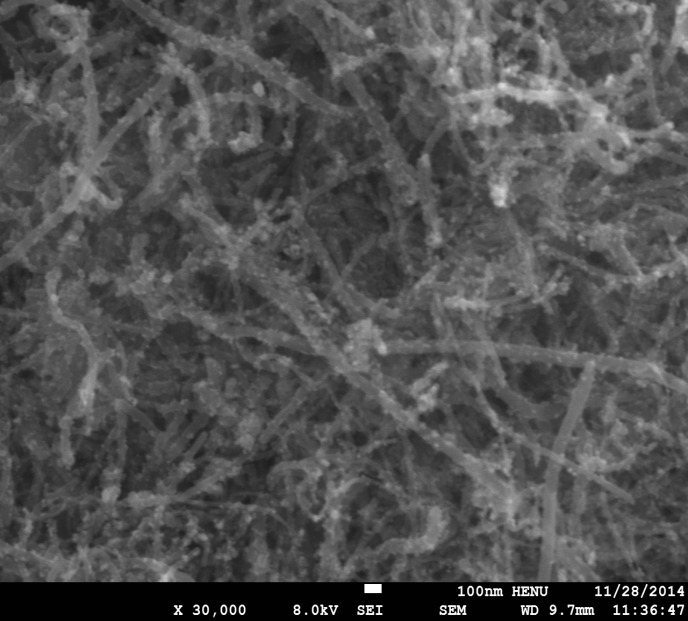


**1 μm**

**(d)**

**(a)**

**(b)**

**(c)**

Figure S1. SEM images of (a) Co_3_O_4_-0.125/MWCNTs; (b) Co_3_O_4_-0.25/MWCNTs; (c) Co_3_O_4_-0.5/MWCNTs; (d) Co_3_O_4_-0.1/MWCNTs
